# Formulation of *Trichoderma sp*. SL2 inoculants using different carriers for soil treatment in rice seedling growth

**DOI:** 10.1186/2193-1801-3-532

**Published:** 2014-09-16

**Authors:** Febri Doni, Anizan Isahak, Che Radziah Che Mohd Zain, Salwati Mohd Ariffin, Wan Nur’ashiqin Wan Mohamad, Wan Mohtar Wan Yusoff

**Affiliations:** School of Biosciences and Biotechnology, Faculty of Science and Technology, Universiti Kebangsaan Malaysia, 43600 Bangi, Selangor D.E, Malaysia; School of Environmental and Natural Resources Sciences, Faculty of Science and Technology, Universiti Kebangsaan Malaysia, 43600 Bangi, Selangor D.E, Malaysia; Paddytech Resources, 22000 Jerteh, Terengganu D.I, Malaysia; Centre for General Studies, Universiti Kebangsaan Malaysia, 43600 Bangi, Selangor D.E, Malaysia

**Keywords:** *Trichoderma sp.*, Rice growth, Corn, Sugarcane bagasse, Inoculant carrier

## Abstract

**Background:**

*Trichoderma sp*. SL2 has been previously reported to enhance rice germination, vigour, growth and physiological characteristics. The use of Potato Dextrose Agar as carrier of *Trichoderma sp*. SL2 inoculant is not practical for field application due to its short shelf life and high cost. This study focuses on the use of corn and sugarcane bagasse as potential carriers for *Trichoderma sp*. SL2 inoculants.

**Findings:**

A completely randomized design was applied for this study. *Trichoderma sp*. SL2 suspension mixed with corn and sugarcane bagasse were used as treatment mixture in soil. Growth parameters including rice seedling height, root length, wet weight, leaf number and biomass were measured and compared to control. The results showed that *Trichoderma sp*. SL2 mixed with corn significantly enhanced rice seedlings root length, wet weight and biomass compared to *Trichoderma sp*. SL2 mixed with sugarcane bagasse and control.

**Conclusion:**

Corn can be a potential carrier for *Trichoderma spp*. inoculants for field application.

## Findings

### Introduction

*Trichoderma spp*. has been exploited as plant growth enhancer and protection against pathogen. *Trichoderma spp*. has been reported as having the potential to act as plant growth promoters to enhance rice growth and productivity (Doni *et al*. 2013). Several phytostimulation mechanisms obtained with *Trichoderma spp.*, including improved root development and auxin production (Contreras-Cornejo *et al*. 2009), siderophore producing (Rawat and Tewari 2011), increased drought tolerance (Shukla *et al*. 2012), expressions of defense protein within the plant (Thakur and Sohal 2013), phosphate-solubilizing (Saravanakumar *et al*. 2013) and increased salt resistance (Contreras-Cornejo *et al*. 2014). Recently, we successfully isolated a local isolate of *Trichoderma spp*. namely *Trichoderma sp*. SL2 which has been proven to enhance rice germination, vigor, seedling growth, vegetative growth, photosynthetic rate, stomatal conductance, internal CO_2_ concentration and water use efficiency (Doni *et al*. 2014a; Doni *et al*. 2014b).

To date the use of *Trichoderma sp*. SL2 is generally in the form of suspension of fungal cells on rice seeds or seedlings. Mass propagation of *Trichoderma sp*. SL2 using solid agar medium in petri dishes could not support large supply of *Trichoderma sp*. SL2. Nakkeeran *et al.* (2005) and Al-Taweil *et al*. (2009) stated that ideal inoculant formulation should have the following criteria; (a) improved shelf life, (b) non-phytotoxic (c) soluble in water and able to release the microbial inoculants with ease, (d) tolerant to bad environment, (e) cost effective and able to control plant diseases (f) and readily available raw material. Therefore, this research was carried out to examine the effectiveness of corn and sugarcane bagasse as *Trichoderma sp*. SL2 carrier and the impact on rice seedling growth.

## Materials and methods

This experiment was conducted at the Fermentation Technology Laboratory and Greenhouse, School of Biosciences and Biotechnology, Faculty of Science and Technology, Universiti Kebangsaan Malaysia. A completely randomized design was performed on this experiment with two treatments and one control; *Trichoderma sp*. SL2 mixed with corn and sugarcane bagasse as carriers and sterilized homogenous sandy clay loam soil without any application as control. Treatments and control were replicated ten times.

### Carriers preparation

Corn and sugarcane bagasse were sterilized by autoclaving at 121°C for 15 minutes. *Trichoderma sp*. SL2 was grown in potato dextrose agar (PDA) and incubated for seven days at 30°C. After incubation, spores of the *Trichoderma spp*. were harvested and diluted with sterilized distilled water until the spore population density reached a value of 10^8^ spore/ml. Ten ml of *Trichoderma sp*. SL2 spore suspension was sprayed to 500 g of the respective carrier, stored in a sterilized polyethylene plastic bag and then incubated for ten days at 30°C. The dosage of *Trichoderma sp*. SL2 formulation was set at 5 g per 1 kg of soil. The inoculated soil was placed in a 15 × 15 cm plastic container.

### Rice seedlings preparation

Rice variety MRQ74 which was previously surface sterilized with 70% ethanol was used for this experiment. The rice seeds were grown in autoclaved homogenous sandy clay under greenhouse condition with ambient temperatures of 26 – 34°C, and placed in a seedling tray. Ten five-day old rice seedlings were grown singly in 15 × 15 cm plastic containers containing each treatment and control. Water was maintained at 2 cm level from the soil surface and actively aerated by physically breaking up the soil surface once every ten days.

### Measurement

Rice seedling growth parameters were measured 15 days after transplanting. Plant height (cm) was measured from ground level to the tip of the longest leaf and leaf number was counted for each treatment and control. Root length (cm) was measured from the base of the stem to the longest root using a ruler and rice seedling wet weight (g) was measured using a digital scale. Rice biomass (g) measurement was done after rice roots were dried in the oven at a temperature of 65°C for seven days.

### Statistical analyses

All data were statistically analyzed using one-way analysis of variance (ANOVA). All treatment means were separated using Fisher’s protected Least Significance Difference (LSD) mean separation at 5% probability level.

## Results and discussion

**Figure 1:**
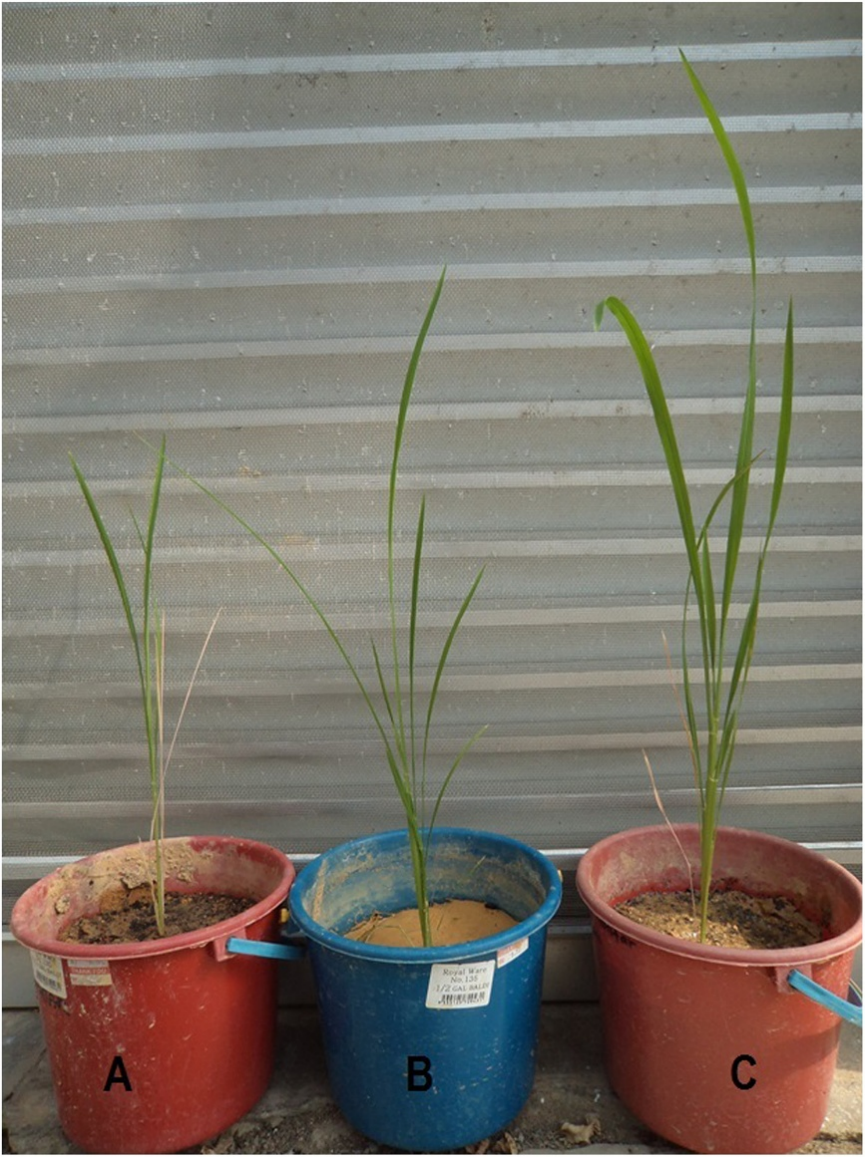
**Rice seedlings treated with (A) control, (B) sugarcane bagasse formulation, (C) corn formulation.**

**Figure 2:**
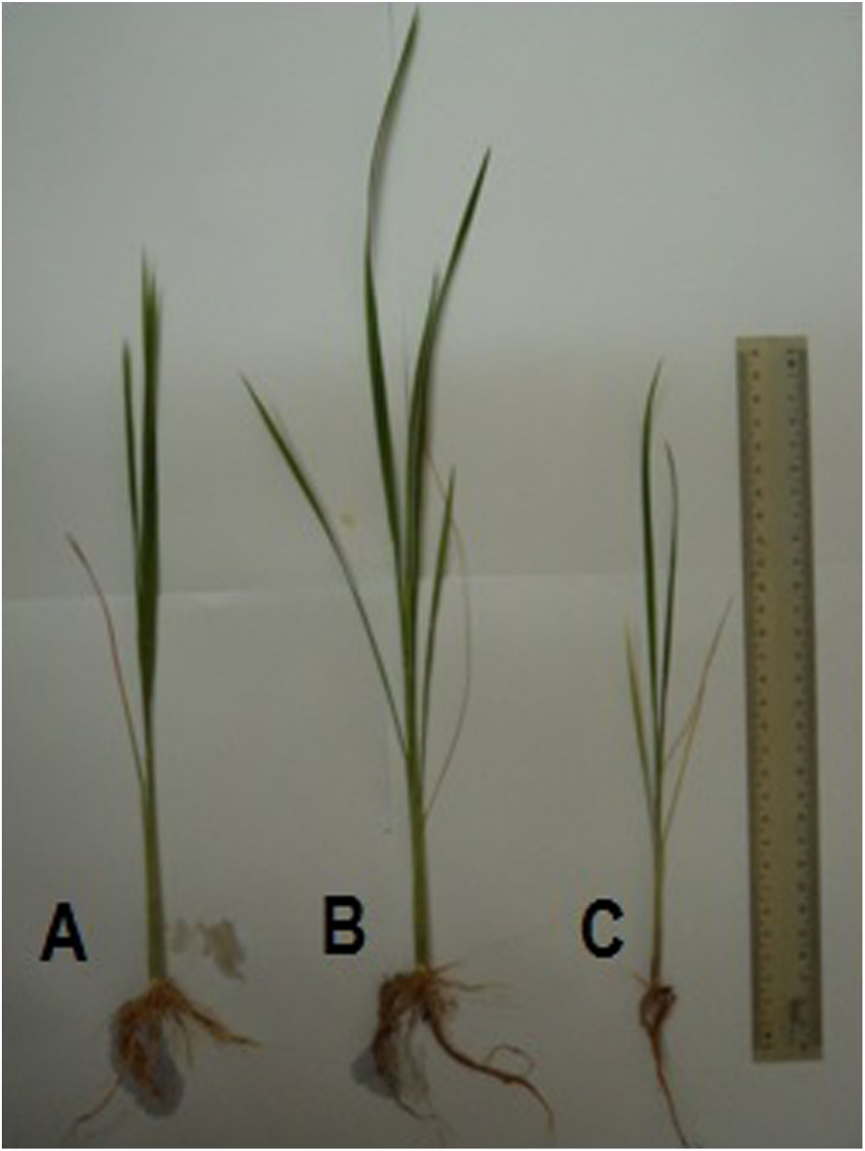
**Rice seedlings treated with (A) sugarcane bagasse formulation, (B) corn formulation, (C) control.**

**Figure 3:**
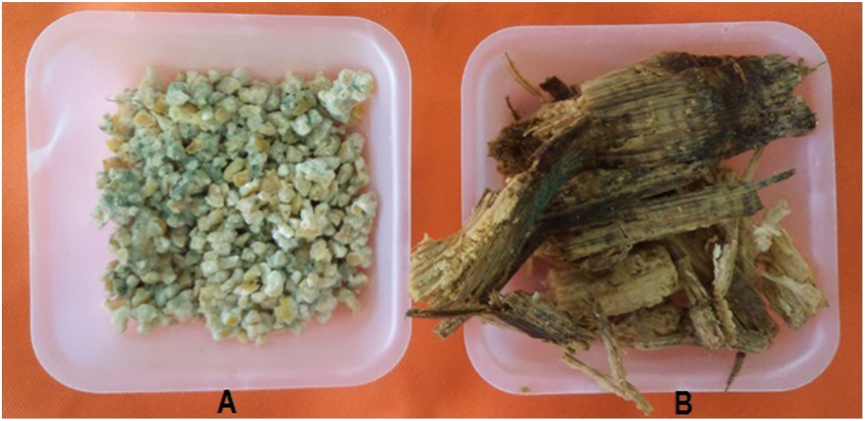
***Trichoderma sp*** **. SL2 grows well on corn (A) and sugarcane bagasse (B) indicated by the green coloration.**

Rice seedling root length, wet weight and biomass treated with *Trichoderma sp*. SL2 -corn carrier was significantly greater than the *Trichoderma sp*. SL2 - sugarcane bagasse carrier and control. However, result was not significant for plant height and leaf number (Table 1, Figures 1 and 2). Corn containing carbohydrate and minerals served as potential carrier for *Trichoderma sp*. SL2 inoculant carrier because it is easily available, stored, commercialized and employed in the field. *Trichoderma sp*. SL2 formulated with corn can be applied in rice cultivation as seed treatment, seed biopriming, root treatment and soil treatment. *Trichoderma sp*. SL2 formulated with corn and sugarcane bagasse showed good growth, color changed to green after ten days *Trichoderma sp*. SL2 treatment (Figure 3). Green coloration of corn and sugarcane bagasse indicated that the fungal mycelium has successful growth.

**Table 1:** **Results of corn and sugarcane bagasse as** ***Trichoderma sp*** **. SL2 carrier on rice seedling growth**

Treatment	Height (cm)	Root length (cm)	Wet weight (g)	Leaf number	Biomass (g)
*Trichoderma sp*. SL2 with corn	32.09 ± 3.68 ns*	12.89 ± 1.68 a**	2.78 ± 0.59 a	6.2 ± 0.78 ns	0.64 ± 0.13 a
*Trichoderma sp*. SL2 with sugarcane bagasse	30.4 ± 3.68 ns	11.1 ± 1.68 a	1.29 ± 0.59 b	5.6 ± 0.78 ns	0.35 ± 0.13 b
Control	26.3 ± 3.68 ns	7.8 ± 1.68 b	0.10 ± 0.59 c	4.8 ± 0.78 ns	0.20 ± 0.13 c

*ns = Not Significant.

**Means with the same letters within the column do not differ significantly according to LSD (p < 0.05).

The ability of *Trichoderma spp*. in enhancing rice plant growth can be employed by several growth promoting mechanisms which include enhancing nutrient availability through solubilization and chelation of minerals (Harman *et al*. 2004), producing phytohormone (Chowdappa *et al*. 2013), releasing elicitors (Nawrocka and Malolepsza 2013) and producing harzianolide (Cai *et al*. 2013). In this research, these mechanisms provided by *Trichoderma* inoculants are believed to be contributing factors that led to better rice plant growth.

## Conclusion

*Trichoderma sp*. SL2 formulated with corn as carrier significantly increased rice seedling growth, thus making corn as a potential carrier to be employed as *Trichoderma spp*. inoculants for field application.

## References

[CR1] Al-Taweil HIB, Osman M, Hamid AA, Wan Mohtar WY (2009). Development of microbial inoculants and the impact of soil application on rice seedlings growth. Am J Agri biol Sci.

[CR2] Cai F, Yu G, Wang P, Wei Z, Fu L, Shen Q, Chen W (2013). Harzianolide, a novel plant growth regulator and systemic resistance elicitor from *Trichoderma harzianum*. Plant Physiol Biochem.

[CR3] Chowdappa P, Kumar SPM, Lakshmi MJ, Upreti KK (2013). Growth stimulation and induction of systemic resistance in tomato against early and late blight by *Bacillus subtilis* OTPB1 or *Trichoderma harzianum* OTPB3. Biol Control.

[CR4] Contreras-Cornejo HA, Macías-Rodríguez L, Cortés-Penagos C, López-Bucio J (2009). *Trichoderma virens*, a plant beneficial fungus, enhances biomass production and promotes lateral root growth through an auxin-dependent mechanism in Arabidopsis. Plant Physiol.

[CR5] Contreras-Cornejo HA, Macías-Rodríguez L, Alfaro-Cuevas R, Lopez-Bucio (2014). *Trichoderma spp*. Improve growth of Arabidopsis seedlings under salt stress through enhanced root development, osmolite production, and Na^+^ elimination through root exudates. Mol Plant Microbe Interact.

[CR6] Doni F, Al-Shorgani NKN, Tibin EMM, Abuelhassan NN, Anizan I, Che Radziah CMZ, Wan Mohtar WY (2013). Microbial involvement in growth of paddy. Curr Res J Biol Sci.

[CR7] Doni F, Anizan I, Che Radziah CMZ, Salman AH, Rodzihan MH, Wan Mohtar WY (2014). Enhancement of rice seed germination and vigour by *Trichoderma spp*. Res J App Sci Eng Technol.

[CR8] Doni F, Anizan I, Che Radziah CMZ, Wan Mohtar WY (2014). Physiological and growth response of rice (*Oryza sativa* L.) plants to *Trichoderma spp*. inoculants. AMB Express.

[CR9] Harman GE, Petzoldt R, Comis A, Chen J (2004). Interactions between *Trichoderma harzianum* strain T22 and maize inbred line Mo17 and effect of this interaction on diseases caused by *Pythium ultmum* and *Colletotricum graminicola*. Phytopathol.

[CR10] Nakkeeran S, Fernando WGD, Siddiqui ZA, Siddiqui ZA (2005). Plant Growth Promoting Rhizobacteria Formulations and its Scope in Commercialization for the Management of Pests and Diseases. PGPR: Biocontrol and Biofertilization.

[CR11] Nawrocka J, Malolepsza U (2013). Diversity in plant systemic resistance induced by Trichoderma. Biol Control.

[CR12] Rawat R, Tewari L (2011). Effect of abiotic stress on phosphate solubilization by Biocontrol Fungus *Trichoderma sp*. Curr Microbiol.

[CR13] Saravanakumar K, Shanmuga Arasu V, Kathiresan K (2013). Effect of *Trichoderma* on soil phosphate solubilization and growth improvement of *Avicennia marina*. Aquat Bot.

[CR14] Shukla N, Awasthi RP, Rawat L, Kumar J (2012). Biochemical and physiological responses of rice (*Oryza sativa* L.) as influenced by *Trichoderma harzianum* under drought stress. Plant Physiol Biochem.

[CR15] Thakur M, Sohal BS (2013). Role of elicitors in inducing resistance in plants against pathogen infection: a review. ISRN Biochem.

